# A comparison of the characteristics, motivations, preferences and expectations of men donating sperm online or through a sperm bank

**DOI:** 10.1093/humrep/dez173

**Published:** 2019-11-11

**Authors:** S Graham, T Freeman, V Jadva

**Affiliations:** Centre for Family Research, University of Cambridge, Free School Lane, Cambridge CB2 3RF, United Kingdom

**Keywords:** sperm donor, donor conception, online connection website, identity-release donation, sperm bank donors, clinic donors, internet, donor

## Abstract

**STUDY QUESTION:**

How do the demographic characteristics, motivations, experiences and expectations of unregulated sperm donors (men donating sperm online through a connection website) compare to sperm donors in the regulated sector (men donating through a registered UK sperm bank)?

**SUMMARY ANSWER:**

Online donors were more likely to be older, married and have children of their own than sperm bank donors, were more varied in their preferences and expectations of sperm donation, and had more concerns about being a sperm donor.

**WHAT IS KNOWN ALREADY:**

While studies have examined motivations and experiences of both regulated sperm bank, and unregulated online sperm donors, no study has directly compared these two groups of donors.

**STUDY DESIGN, SIZE, DURATION:**

An email was sent to the 576 men who were registered sperm donors at the London Sperm Bank, the UK’s largest sperm bank regulated by the Human Fertilisation and Embryology Authority (HFEA), who had commenced donation between January 2010 and December 2016, and had consented to be contacted for research. The online survey, which contained multiple choice and open-ended questions, was completed by 168 men over a 7-week period. The responses were compared to those of sperm donors registered on Pride Angel, a large UK-based connection website for donors and recipients of sperm: our research team had already collected these data. In total, 5299 sperm donors were on Pride Angel at time of data capture and 400 men had completed a similar survey. The responses of 70 *actual* online sperm donors (i.e. those whose sperm had been used to conceive at least one child) were used for comparison with the sperm bank donors.

**PARTICIPANTS/MATERIALS, SETTING, METHODS:**

The survey obtained data on the sperm donors’ demographic characteristics, motivations, experiences and expectations of sperm donation. Data from sperm bank donors were compared to online donors to examine differences between the two groups. The study compared online and clinic donors who had all been accepted as sperm donors: online donors who had been ‘vetted’ by recipients and sperm bank donors who had passed the rigorous screening criteria set by the clinic.

**MAIN RESULTS AND THE ROLE OF CHANCE:**

A response rate of 29% was obtained from the sperm bank donors. Online donors were significantly older than sperm bank donors (mean ± SD: 38.7 ± 8.4 versus 32.9 ± 6.8 years, respectively) and were more likely to have their own children (p < 0.001 for both characteristics). Both groups rated the motivation ‘I want to help others’ as very important. Online donors rated ‘I don’t want to have children myself’, ‘to have children/procreate’ and ‘to enable others to enjoy parenting as I have myself’ as more important than sperm bank donors, whereas sperm bank donors rated financial payment as more important than online donors, as well as confirmation of own fertility. Most (93.9%) online donors had donated their sperm elsewhere, through other connection sites, fertility clinics, sperm banks or friends and family, compared to only 2.4% of sperm bank donors (p < 0.001). There was a significant difference in how donors viewed their relationship to the child, with online donors much less likely than sperm bank donors to see their relationship as a ‘genetic relationship only’. Online donors had more concerns about being a donor (p < 0.001), for example, being concerned about ‘legal uncertainty and child financial support’ and ‘future contact and uncertainty about relationship with donor-conceived child’.

**LIMITATIONS, REASONS FOR CAUTION:**

Findings may not be representative of all sperm donors as only one online connection site and one HFEA registered sperm bank were used for recruitment.

**WIDER IMPLICATIONS OF THE FINDINGS:**

Despite concern regarding shortages of sperm donors in licensed clinics and unease regarding the growing popularity of unregulated connection websites, this is the first study to directly compare online and sperm bank donors. It highlights the importance of considering ways to incorporate unregulated online sperm donors into the regulated sector. With many online donors well aware of the legal risks they undertake when donating in the unregulated online market, this would both increase the number of sperm donors available at clinics but also provide legal protection and support for donors.

**STUDY FUNDING/COMPETING INTEREST(S):**

This study was supported by the Wellcome Trust Grants 104 385/Z/14/Z and 097857/Z/11/Z. The authors have no conflicts of interest.

## Introduction

In 2005 the UK removed donor anonymity meaning that all sperm donors who donate through a clinic or sperm bank registered with the Human Fertilisation and Embryology Authority (HFEA) agree to their identifying information being released to individuals born from their donation after they reach 18 years of age (HFEA, 2004). At the time the legislation came into force, there was concern about the possible effect this may have on donor numbers. Over half of the 43 UK sperm donors studied by Frith et al. (2007) stated that they would not continue donating if anonymity were removed. In reality donor numbers increased rapidly after a short initial decline (Day, 2007), and some clinics with active recruitment programmes have been successful in finding sufficient donors (Blyth and Frith, 2008; Ahuja, 2011). However, demand for donors has continued to outstrip supply and there has been a steady increase in the number of UK clinics importing sperm from overseas to meet demand. By 2013, imported sperm made up almost one-third of new sperm donor registrations (HFEA, 2014). In 2014, the National Sperm Bank, a joint collaboration between the National Gamete Donation Trust and Birmingham Fertility Centre, was set up (Lea, 2016). It had been funded by a start-up grant from the Department of Health, established to increase the supply of donor sperm in the UK, particularly within National Health Service clinics, aiming to prevent a shortage of donors from driving patients overseas or to unregistered services. However, the National Sperm Bank closed after less than 2 years, having only recruited eight donors.

While discussions regarding the scarcity of HFEA registered sperm donors continued, attention was drawn to the increase in number of men donating their sperm outside of the regulated sector, a phenomenon that has been facilitated by the rise of dedicated websites, often called ‘connection sites’, that can connect recipients and donors directly (Freeman et al., 2016b; Whyte, 2019). Unlike men who donate through clinics and sperm banks, online donors have the opportunity to meet recipients at the outset, and can also have greater control over who they donate to, as well as levels of information exchange and contact (Freeman et al., 2016b; Bossema et al., 2014; Woestenberg et al., 2016).

Connection sites are legal but unregulated (Harper et al., 2017). HFEA registered donors must be aged between 18 and 41 years and undergo medical screening. They receive £35 per clinic visit, plus travel, accommodation and childcare expenses where necessary (HFEA, 2018). In contrast, online donors do not have to meet the criteria relating to age or medical screening required of clinic donors. Whereas HFEA registered donors do not have any legal rights or responsibilities to children conceived from their donation, men donating outside a licensed clinic do not have such protection. The HFEA states that in the UK the law regarding legal parentage is not straightforward if donation takes place through a private arrangement: it is possible that the donor will be the legal father of the child with all the parental and financial responsibility involved (HFEA, 2019). They advise that parental responsibility can depend upon whether the woman is single, married or in a civil partnership at the time of conception; whether the insemination took place through artificial insemination or sexual intercourse; who is named on the birth certificate and whether the donor has established a relationship with the child. They clarify that a donor cannot opt out of being the legal father of the child, even if the mother agrees to that, and any agreement drawn up to that effect has no legal standing.

Online sperm donation has therefore raised concerns about personal, medical and legal risks to all parties involved. The HFEA highlights concerns about the resultant child’s legal parentage and future access to donor information, the number of children created from any one donor, the lack of medical screening of donors and the potential sexual exploitation of recipients (HFEA, 2014). Thus, there have been calls for donors and recipients to have access to clear, accurate information about the implications of informal sperm donation (Harper et al., 2017).

A systematic review of the literature regarding sperm donors (incorporating known and anonymous, commercial and volunteer populations) identified four different types of donor motivation; altruism, financial compensation, procreation and confirmation of own fertility status (Van den Broeck, 2013). Similarly, online donors have been reported to donate for altruistic reasons although procreation was also viewed as important (Freeman et al., 2016b; Woestenburg et al., 2016). Online donation also enables donors to donate ‘anonymously’ whereby recipients do not disclose the donor’s identity to the resultant child, and in some cases to the recipient (Freeman et al., 2016b). Donation preferences have been found to differ by sexual orientation, with heterosexual men more likely to seek anonymous donation compared to gay and bisexual men who favour known donation (Freeman et al., 2016b). The different routes to donation raise questions about how online donors may differ from HFEA registered donors, and whether they have different views about donation and about their involvement with the resultant child. A UK study of online donors which compared the experiences of exclusively online donors to online donors who had also donated through a clinic found that exclusively online donors were more likely to be in a committed relationship and were more likely to identify as gay, bisexual or asexual (Whyte, 2019). However, no studies have directly compared HFEA registered donors with those who donate through internet sites.

The present study compares the characteristics, motivations, experiences and expectations of donors who have who have donated in the unregulated sector via an online connection site to donors who have donated sperm in the regulated sector through an HFEA registered sperm bank. With large numbers of men registering to be online donors, and concerns about meeting growing demand in the regulated sector, a comparison between these two groups of sperm donors will elucidate similarities and differences between them. Such information will be of value to policy makers and clinicians alike, enabling an exploration of whether there are lessons to be learnt from online donation that might shed light on how to increase donor numbers in the regulated sector.

## Materials and Methods

Donors were recruited to this study from the London Sperm Bank, the UK’s leading regulated provider of donor sperm. An email was sent to the 576 men who had become HFEA-registered sperm donors, commenced a donation programme at London Sperm Bank between January 2010 and December 2016, and had consented to be contacted for research. The email, sent by staff at the London Sperm Bank, contained a link to the survey as well as information about consent procedures. It was followed up where applicable by two reminder emails. All donors who completed the survey were eligible to claim a £15 Amazon Voucher. The survey was live for 7 weeks between January and March 2017. In total, 168 donors completed the survey, giving a response rate of 29%.

Data for the comparison group of online sperm donors had already been collected by the research team (Freeman et al., 2016b). Online donors registered with the website Pride Angel, one of the largest and well-known online connection sites in the UK, had completed a similar survey between February and March 2014. Further details of the recruitment of online sperm donors are reported in Freeman et al., (2016b). There were 5299 sperm donors registered on Pride Angel at the time of data capture. A total of 400 sperm donors completed the survey. The responses of the 70 *actual* online sperm donors—those whose sperm had been used to conceive at least one child—were used for comparison with the sperm bank donors. Men who have been accepted as sperm bank donors donate in the expectation that their sperm will be used for treatment of recipients and that children will be conceived. Therefore for both groups, thoughts and feelings about sperm donation, and their connection, if any, with recipients of, and offspring conceived with, their sperm, are grounded in the context of a child being conceived as a direct result of their donation. Freeman et al., (2016b) found that there were differences between actual online donors (those who had conceived a child) and the larger pool of online donors who were registered on the site. A greater proportion of actual donors were UK residents, white, in employment and reported having children of their own, when compared with the potential donors registered on the site (Freeman et al., 2016b). Moreover, Freeman stated that recipients ‘filter out’ donors who are less well intentioned. For example, although almost half (44.1%) of the potential online donors had stated a preference for donating through natural insemination, the large majority (94.3%) of actual donors had donated by artificial insemination. Jadva et al (2018) report recipients describing ‘dishonest donors’—men whose motivations were unclear, who did not respond to messages or who were looking for sex—as a disadvantage of connection sites. Recipients on connection sites therefore engage in a process of ‘vetting’ sperm donors for suitability (Jadva et al. 2018). The current study therefore aimed to compare online and clinic donors who had all been accepted as sperm donors—online donors who had been ‘vetted’ by recipients, and sperm bank donors who had passed the rigorous screening criteria set by the clinic.

### Measures

The survey for sperm bank donors was based on the survey completed by the online donors and contained both multiple choice and open ended questions. Additional sections were added to capture clinic-specific experiences.

Data were obtained on:—*Socio-demographic characteristics*: age, sexual orientation, relationship status, parental status, country of residence, ethnicity, education and employment status.—*Motivations for donating sperm*: participants were asked to rate various motivations, e.g. ‘To do something valuable and worthwhile’ or ‘my sperm would go to waste otherwise’ on a scale ranging from 1 ‘not at all important’ to 5 ‘very important’.—*Experiences of donating sperm*: participants were asked who they had discussed their donation with and, if applicable, their partners feelings about donation (rated as either positive, neutral, negative, mixed, not sure), whether they had any concerns about being a donor (4 point scale range from 1 ‘no concerns’ to 4 ‘major concerns’) and if yes, what these concerns were (open-ended response).—*Expectations of sperm donation*: participants were asked about their preferred type of donation (e.g. anonymous, identity-release, known, co-parent), whether or not they would like to place restrictions on who can use their sperm (yes/no/not sure), whether there should be a limit on the number of children born from their donation (yes/no/not sure), if they desired contact with the child (yes/no/not sure), how they viewed their relationship with the child (e.g. no relationship, a genetic relationship only), whether the child should be told about their donor conception (yes/no/not sure), and whether the child should be told the donor’s identity (yes/no/not sure).

The survey for clinic donors was piloted to ensure questions were meaningful and to check survey functionality.

### Data Analysis

Quantitative data were analysed using Student’s t-tests for normally distributed data and Mann Whitney *U* Tests for data not normally distributed. Categorical data were analysed using Chi-square and Fisher’s Exact tests. A P-value of less than 0.05 was considered statistically significant. IBM SPSS Statistics for Windows, Version 25.0, Armonk, NY: IBM Corp, was used for data analysis. Responses to open-ended questions about donors’ concerns regarding being a sperm donor were systematically categorised into themes and are presented as number of cases and percentages.

### Ethics approval

Ethical approval for this study was obtained from the University of Cambridge Psychology Research Ethics Committee. Participant consent was obtained electronically prior to them completing the online survey.

## Results

### Demographic characteristics

Table I shows the demographic characteristics for both groups. There was a significant difference in the age of donors with online donors being significantly older (Mean = 38.7, SD =8.4) than the sperm bank donors (Mean = 32.9, SD =6.8) (*t*(108) = −5.05, *p* = <.001) . Differences were found between type of donor and relationship status (Fisher’s exact = 0.000), with online donors more likely to be married or in a civil partnership and sperm bank donors more likely to be in a cohabiting relationship or to have a non-cohabiting partner. Online donors were much more likely to have their own children compared to sperm bank donors, with 74.3% (52) of online donors having children compared to 17.3% (29) of sperm bank donors (χ^2^(1) = 71.57, p < 0.001). This difference was still significant when online donors were compared to sperm bank donors within the same age range. A total of 50 (71.4%) of the online donors were based in the UK at the time of completing the survey, compared to 150 (89.3%) sperm bank donors. A total of 42 (60%) online donors stated their nationality was British, compared to 112 (66.7%) sperm bank donors. Both groups were therefore international populations. There were no differences in ethnicity, sexual orientation or employment status between the two groups.

**Table I:** Socio-demographic characteristics of sperm donors.

	Sperm bank donors	Online donors	
	Mean (SD)	Mean (SD)	*p*
Age (years)	32.9 (6.8)	38.7 (8.4)	<.001^a^
			
	N (%)	N (%)	
Ethnicity			ns
White	144 (85.7)	62 (88.6)	
Black	3 (1.8)	3 (4.3)	
Asian	11 (6.5)	3 (4.3)	
Mixed race	5 (3.0)	2 (3.9)	
Other	5 (3.0)	0 (0)	
			
Education			.000^c^
Less than secondary school	0 (0)	3 (4.3)	
Secondary school	4 (2.4)	3 (4.3)	
College or trade qualification	25 (14.9)	22 (31.4)	
University degree or higher	137 (81.5)	42 (60)	
Not specified	2 (1.2)	0 (0)	
			
Sexual orientation			ns
Heterosexual	116 (69)	58 (82.9)	
Gay	38 (22.6)	8 (11.4)	
Bisexual	9 (5.4)	4 (5.7)	
Other	5 (3.0)	0(0)	
			
			
Relationship status			.000^c^
Single			
Single	61 (36.3)	30 (42.9)	
Divorced/Separated	7 (4.2)	4 (5.7)	
Widowed	0 (0)	1 (1.4)	
Partnered			
Married/civil partnership	31 (18.5)	25 (35.7)	
Cohabiting	42 (25)	10 (14.3)	
Non-cohabiting partner	26 (15.5)	0 (0)	
			
			
Employment status			ns
Employed full-time	128 (76.2)	60 (85.7)	
Employed part-time	23 (13.7)	4 (5.7)	
Not employed	17 (10.1)	5 (7.1)	<.001^b^
Not specified	0 (0)	1 (1.4)	
			
Donated sperm elsewhere			
Yes	4 (2.4)	62 (88.6)	
No	164 (97.6)	7 (10)	
Not specified	0 (0)	1 (1.4)	

a Student t-test

b Chi-square-test

c Fishers exact test

### Motivations for donating sperm

The majority of both online and sperm bank donors rated the motivation ‘I want to help others’ as ‘very important’ (63.1%, n = 106 of sperm bank donors and 87.1%, n = 61 of online donors) (Table II). However, online donors rated the following motivations as more important than sperm bank donors: ‘I don’t want to have children myself’ (*U* = 1289, *z* = −3.33, *p* = 0.001), ‘want to help others’ (*U* = 4217, *z* = −3.91, *p* < 0.001), ‘to enable others to enjoy parenting as I have myself’ (*U* = 1107, *z* = −2.77, *p* = 0.006), ‘to have children/procreate’ (*U* = 3814, *z* = −2.537, *p* = 0.011), ‘to do something valuable and worthwhile’ (*U* = 4095.5, *z* = −3.62, *p* < 0.001) and ‘my sperm would go to waste otherwise’ (*U* = 2737, *z* = −3.62, *p* < 0.001). Sperm bank donors rated ‘financial payment’ as more important than online donors (*U* = 1433, z = −3.465, p = 0.001) as well as ‘confirmation of own fertility’ (*U* = 3005, *z* = −1.97, *p* = 0.048). No other differences were found. See Table II for medians and interquartile range for both groups.

**Table II:** Motivations for donating sperm.

	Sperm bank Donors	Online Donors	
	**Median (Interquartile Range)**	**Median (Interquartile Range)**	*p*
Motivations^*^			
I don’t want to have children myself	2 (3)	3 (3)	.001
Want to help others	5 (1)	5 (0)	.001
To enable others to enjoy parenting as I have myself	4 (2)	5 (1)	.006
Financial payment	3 (2)	1 (2)	.001
Confirmation of my own fertility	4 (1)	3 (4)	.048
To pass on my genes	4 (2)	4 (1)	ns
To have children/procreate	4 (3)	4 (2)	.011
Personal experience of infertility^a^	2 (3)	3 (4)	ns
Knowledge of infertility amongst family/friends^b^	3.5 (2)	3 (3)	ns
Knowledge of egg or sperm donation amongst family/friends^c^	3 (3)	3 (3)	ns
To do something valuable and worthwhile	4 (1)	5 (1)	<.001
My sperm would go to waste otherwise	3 (3)	4 (2)	<.001
No reason not to	3 (1)	4 (2)	ns
Other reason	2 (1)	4 (4)	ns

Mann Whitney U tests conducted

^a^The option given to online donors was ‘My partner is infertile or has fertility problems’

^b^The option given to online donors was ‘Family/friends have experienced infertility’

^c^The option given to online donors was ‘Family/friends have used sperm or egg donation’

^*^Scale ranged from 1 ‘not at all important’ to 5 ‘very important’

### Experience of donation

Most (88.6%, n = 62) online donors had donated their sperm elsewhere compared to 2.4% (n = 4) of sperm bank donors (χ^2^(1) = 186.26, *p* < 0.001). Of the 62 online donors who had previously donated sperm, 43 (69.4%) had previously donated on Pride Angel, 38 (61.3%) through another connection site, 12 (19.4%) through a fertility clinic, four (6.4%) through a sperm bank, nine (14.5%) to a friend and two (3.2%) to a family member.

Table III shows who donors had discussed their donation with, and the reactions of their partners, if told. Overall, most donors had not discussed their donation with others, and for both groups the people they discussed their donation with the most were their friends, and their partner. Online donors were more likely than sperm bank donors to have discussed their donation with other family members (Fisher’s Exact = 0.045), friends (χ^2^(1) = 8.81, *p* = 0.005), work colleagues (Fisher’s Exact = 0.002), doctors or other professionals (Fisher’s Exact = 0.000), and a counsellor or psychologist (Fisher’s Exact = 0.001). For donors who had discussed their donation with their partners, the largest proportion (40%, n = 25, of clinic donors and 40.6%, n = 13, of online donors) reported their partners to have felt positively about it, with no differences found between the groups in how their partners responded.

**Table III:** Experience of donation.

	Sperm bank donors	Online donors	
	N (%)	N (%)	*p*
Concerns about being a donor			<.001^a^
No concerns	134 (79.8)	34 (48.6)	
Yes, minor concerns	28 (16.7)	24 (34.3)	
Yes, moderate concerns	3 (1.8)	9 (12.9)	
Yes, major concerns	1 (0.6)	2 (2.9)	
Not specified		1 (1.4)	
Discussion with others			
I would tell anyone	9 (5.4)	6 (8.6)	ns
My spouse/partner	49 (29.2)	22 (31.4)	ns
My children	0 (0)	3 (4.3)	ns
My mother	26 (15.5)	11 (15.7)	ns
My father	20 (11.9)	11 (4.6)	ns
My siblings	16 (9.5)	9 (12.9)	ns
Other family members	5 (3.0)	7 (10.0)	.045^b^
Friends	50 (29.8)	35 (50)	.005^c^
Work colleagues	5 (3.0)	10 (14.3)	.002^b^
Doctor or other health professional	2 (1.2)	10 (14.3)	.000^b^
Counsellor or psychologist	2 (1.2)	8 (11.4)	.001^b^
Other	2 (1.2)	1 (1.4)	ns
Spouse/partner’s feelings about donation			ns
Positive	25 (40)	13 (40.6)	
Neutral	13 (20)	4 (12.5)	
Negative	9 (13.8)	6 (18.8)	
Mixed	13 (20)	7 (21.9)	
Not sure	4 (6.2)	2 (6.3)	

^a^Mann Whitney U test

^b^Fisher’s Exact test

^c^Chi-Square test

Online donors had more concerns (median = 2, IQR = 1) about being a donor compared to sperm bank donors (median = 1, IQR = 0) (U = 3821.5, z = −5.084, *p* < 0.001). Twenty-six online donors and 29 sperm bank donors elaborated on their concerns in the free text option. Online donors’ concerns centred around the themes of ‘legal uncertainty and child financial support’ (38%, n = 10), ‘future contact and uncertainly about relationship with donor-conceived child’ (31%, n = 8), ‘secrecy and impact of donation on own family’ (27%, n = 7), ‘the child – their health, happiness and the donor’s feelings of responsibility towards them’ (n = 4) and ‘the donation process’ (15%, n = 4). Sperm bank donors’ concerns were on the themes of ‘Identity-release and contact with donor-conceived offspring at that time’ (45%, n = 13), ‘the child – their health, happiness and the donor’s feelings of responsibility towards them’ (31%, n = 9), ‘discussing sperm donation with and its impact on others’ (17%, n = 5) and ‘lack of information regarding donation’ (17%, n = 5).

### Expectations of donation

Responses to questions regarding the donors’ expectations and preferences regarding sperm donation can be seen in Table IV. There was a significant difference in the preferred type of donation (Fisher’s exact <.001), with sperm bank donors showing a preference for identity-release donation (donor’s identity can be accessed by child at age 18 years) and online donors preferring anonymous (donor’s identity remains unknown) or known (donor’s identity is known from the outset) donation.

**Table IV:** Expectations of donation.

	Sperm bank donors	Online donors	
	n (%)	n (%)	*p*
Preferred type of donation			
Anonymous	25 (14.9)	18 (25.7)	.001^a^
Identity release	100 (58.5)	17 (24.3)	
Known	18 (10.7)	27 (38.6)	
Co-parent	7 (4.2)	4 (5.7)	
Other/don’t know	18 (10.7)	4 (5.7)	
Not specified	0 (0)	0 (0)	
Ideally, would you like to place restrictions on who could use your donated sperm?^*^			
Yes	29 (17.3)	44 (62.9)	<.001^b^
No	118 (70.2)	14 (20)	
Not sure	19 (11.3)	12 (17)	
Not specified	2 (1.2)	0 (0)	
Should there be a limit on the number of children that can be born from the sperm of any one donor?			
Yes	69 (41.3)	32 (45,7)	ns
No	61 (36.5)	26 (37.1)	
Not sure	37 (22.2)	12 (17.1)	
Future contact with child			
Yes	61 (36.7)	34 (48.6)	ns
No	41 (24.7)	18 (25.7)	
Not sure	64 (38.6)	18 (25.7)	
View of relationship with child			
No relationship	15 (8.9)	14 (20.0)	.031^a^
A ‘genetic’ relationship only	59 (35.1)	13 (18.6)	
Like any other child I know	12 (7.1)	3 (4.3)	
Like a friend’s child	15 (8.9)	10 (14.3)	
Like a niece or nephew	12 (7.1)	9 (12.9)	
A special relationship	38 (22.6)	13 (18.6)	
Like my own child	15 (8.9)	7 (10)	
Not specified	2 (1.2)	1 (1.4)	
Disclosure about donor conception			
Yes	71 (42.3)	37 (52.9)	ns
No	4 (2.4)	3 (4.3)	
Not sure	9 (5.4)	6 (8.6)	
It’s up to parents	83 (49.4)	24 (34.3)	
Missing	1 (.6)	0 (0)	
Disclosure about donor identity			.015^b^
Yes	61 (36.3)	26 (37.1)	
No	12 (7.1)	15 (21.4)	
Not sure	22 (13.1)	7 (10)	
It’s up to parents	70 (41.7)	22 (31.4)	
Missing	3 (1.8)	0 (0)	

^*^Question asked to online donors was ‘Do you have any restrictions about who you would donate your sperm to?’

^a^Fisher’s Exact test

^b^Chi-Square test

Online donors were more likely to want to place restrictions on who could use their donated sperm (62.9%, n = 44 of online donors compared to 17.3%, n = 29 of sperm bank donors) (χ^2^(2) = 56.98, *p* < 0.001). Online donors and sperm bank donors did not differ in whether there should be a limit placed on the number of children born from their donation. There was no significant difference by type of donor in expecting contact with the donor-conceived child with almost half (48.6%, n = 34) of online donors expecting future contact compared with 36.7% (n = 61) of sperm bank donors. However, there was a significant difference in how donors viewed their relationship with the child, with sperm bank donors much more likely than online donors to see their relationship as a ‘genetic relationship only’ (Fisher’s exact = 0.031). There was no significant difference between online donors and sperm bank donors in their responses to whether children should be told about their conception, with 52.9% (n = 37) of online donors responding that donor-conceived children should be told about their donor conception compared to 42.3% (n = 71) of sperm bank donors. However, online donors were more likely than sperm bank donors to respond ‘no’ to whether they thought the child should be told their donor’s identity, with 21.4% (n = 15) of online donors stating that donor-conceived children should not be told about their donor’s identity, compared to 7.1% (n = 12) of sperm bank donors (χ^2^(3) = 10.53, *p* = 0.015).

## Discussion

When donor anonymity was removed in 2005, it was speculated there would be a shift in the demographics of HFEA registered sperm donors to an ‘older, married donor who is likely to have their own children’ (Day, 2007). However, this study has found that it is online donors, not HFEA registered donors, who are more likely to fit this demographic. Online donors were older than sperm bank donors and more likely to be married or in a civil partnership with children.

The current age limit for HFEA registered donors in the UK is 41 years. Data from the HFEA, however, indicate that many donors surpass this upper age limit: in 2013 a quarter of donors were aged over 40 years (HFEA, 2013), the majority of whom are likely to be ‘known’ donors, although no distinction is made between previously known donors, such as family or friends, and donors who recipients may have met through a third party such as a connection site. Recently there has been interest in the impact of increased paternal age on fertility outcomes and child health. Ghuman et al. (2016), however, found that live birth and miscarriage rates following ART using donor sperm were not affected by the age of the sperm donor, up to the age of 45 years. There are clearly older men who already have their own children who are willing to donate sperm on online connection sites and recipients who are happy to use this sperm. Perhaps raising the age limit for donors in the regulated sector would enable more men to donate via this route and increase donor numbers.

Over one-quarter of sperm bank donors who stated they had a partner had not discussed being a sperm donor with them, echoing previous research indicating that only a minority of donors involve their partners in the decision-making process (Van den Broeck et al., 2013). These sperm donors will need to decide whether to disclose their role as a donor to this partner, any future partner, as well as any children they may have in the future. Daniels et al. (2005) describe how anonymous sperm donors’ thoughts and feelings about their donation changed over time. The experience of bringing up children was one of the reasons given by anonymous sperm donors to later prefer identity-release donation (Daniels et al., 2005), a phenomenon also noted by Kirkman et al. (2014). Transition to parenthood may therefore alter sperm donors’ thoughts and feelings about being a donor and future information exchange and contact with donor-conceived children. Online donors were more likely to already have children of their own before becoming a sperm donor and were more open about their donation with others. It is possible that being a parent may make them more likely to want to share information about themselves with their donor offspring, a desire which online donation enables them to fulfill.

As in previous studies of sperm donors (see Van den Broek et al., 2013), this study has shown that motivation to donate, either online or through a sperm bank, is multifaceted. Differences were found, however, in how important these two groups of donors rated their various motivations. Motivations concerning parenting and procreation were more important for online donors than sperm bank donors. This finding perhaps indicates the plurality of meanings that being a sperm donor can entail for online donors: although connection site donors are often referred to as known donors, ‘known’ donation in this context exists on a continuum ranging from minimal contact to co-parenting (Freeman et al., 2016b; Ravelingien, 2017; Dempsey, 2010; Goldberg & Allen, 2013; Jadva et al., 2018). The flexibility in donation practices and the levels of donor involvement and information exchange that could take place between donors, recipients and donor-conceived offspring, allow for multiple, and at times seemingly contradictory, motivations to donate. For example, online donation could allow some donors to fulfil the motivation, ‘I want to procreate/have children’, whilst for others it could fit with their view that they ‘don’t want children’ themselves. These were both motivations that online donors rated more highly than sperm bank donors. What ‘having children’ means for these donors may also vary. For some ‘having children’ may take on a very biological meaning, the process of passing on one’s genes and contributing to reproduction, while for others it may have a more social element, being involved with and helping to raise the child. Online donation could fulfil both these meanings, with donors being able to be anonymous to the donor-conceived child or a part of their life.

‘Financial payment’, often seen as a controversial motivation, was rated as more important by sperm bank donors than online donors. When higher limits for donor compensation were brought into effect by the HFEA in the UK in 2012 they were met with controversy (Wilkinson, 2016), with some being concerned that ‘money detracts from the altruistic nature of donation and that non-altruistic donation may have a negative effect on the welfare of the future child, who may in fact feel that they were “bought”’ (HFEA, 2011: 13, in Wilkinson, 2016). However, others believe that paid donors may be perceived as more suitable donors because they have ‘the right attitudes for the job’ (Pennings, 1997: 1842): they do not wish their involvement to go beyond donation. As can be seen from the findings of this study, and others (Van den Broek et al., 2013), financial payment is only one motive amongst others for sperm bank donors: altruistic and financial motives are not mutually exclusive.

Responses to questions about donor expectations again illustrate the flexibility of what online sperm donation can entail. There was a significant difference in how donors viewed their relationship with a resulting child, with sperm bank donors more likely than online donors to see their relationship as a ‘genetic relationship only.’ Online donors were more evenly split in how they viewed their relationship with the resulting child, perhaps corresponding to how far along the spectrum of ‘donor’ to ‘co-parent’, described by Ravelingien et al. (2016), they wished to be. Conversely, UK sperm bank donors are donating within a very clear policy context of what being a ‘good’ sperm donor entails (Graham, Mohr and Bourne, 2016): the donor has no rights or responsibilities to the child conceived, just a ‘genetic relationship’. It is important to note, however, that once the donor is identifiable to the child there is, as yet, no clear cultural script for how sperm donors should navigate any relationship that may follow. Whether these particular identity-release donors will differ in how they describe their relationship with an 18-year old conceived with their sperm with whom they are in contact is yet to be explored.

Although there was no significant difference between sperm bank and online donors in their responses to whether children should be told about their conception, online donors were more likely to state that a child shouldn’t be told their donor’s identity. Although initially surprising, this is again likely to represent the continuum of online donors’ preferences for information exchange and their relationship with the donor conceived child, with some online donors remaining anonymous to the child (Jadva et al., 2018). Interestingly, there was no significant difference between the two groups in terms of expecting future contact with the child. Of course there may be wide discrepancy between *expected* and *actual* contact and the donor’s views about contact may change over time.

Existing research indicates that gay sperm donors may be more open to contact and information exchange with recipients (Riggs, 2008, Riggs and Russel, 2011, Freeman et al. 2016b). Whyte et al. (2017) found that non- heterosexual donors on average donated less regularly, donated to fewer women, had fewer offspring and had not been donating as long, when compared with the heterosexual online donors. They suggest that this may be because non-heterosexual donors may invest more psychologically, emotionally and financially to the women and couples that they donate to, and are seeking a more co-operative and ongoing interactive arrangement. Given Pride Angel’s open orientation towards the lesbian, gay, bisexual, and transgender (LGBT) community, and online donation’s potential to facilitate more contact between donors and recipients, it is perhaps surprising that no difference was found between the online and sperm bank donors’ sexual orientation. Further research is needed to explore whether any other characteristics affect a donor’s openness to information exchange and contact.

Most online donors had donated sperm elsewhere compared to only a very small minority (2.4%) of sperm bank donors. These findings have implications for the limitations on the number of offspring conceived to any one donor. Media articles have exposed the stories of a handful of online donors who have conceived large numbers of children. One man was reported to have ‘fathered’ over 800 children by ‘selling his sperm for £50 a time online’ (Crocker, 2016). Such large numbers of donor-conceived offspring raise concerns about unintentional consanguinity as well as the psychosocial impact of the discovery of large numbers of genetic half siblings (Freeman, Jadva and Slutsky, 2016a). In the UK, HFEA registered donors’ sperm can only be used to conceive offspring in up to ten families. However, the separation between clinic and online donation may not be discreet: donors may switch from regulated donation to the informal, unregulated sector, and in doing so exceed the policy limits. Although the majority of online donors who had previously donated sperm had done so through Pride Angel or another connection site, over a quarter of these men had donated through a fertility clinic or sperm bank. Whyte (2019) reports on a different survey of 112 men registered on the connection site Pride Angel. Here, 31.6% of the sample had experience of clinical donation prior to participating in the informal market. Informal donors with a history of clinical donation were found to donate more frequently each month and had been donating in the online market for longer than those who had only ever donated informally (Whyte et al., 2017). Such a finding suggests that this particular group of donors may be highly motivated to donate to many recipients. Although data from the current study suggest that very few of the sperm bank donors had gone on to donate sperm in any other way since completing the donation programme (Graham et al, in preparation) it is conceivable that some may do so in the future. It is important that HFEA registered donors are counselled on the reasons for the ten family limit and the potential risks of proceeding to donate sperm online.

Online donors were found to have more concerns about being a donor than sperm bank donors. The most common concern cited in the free text option for online donors centred around the legal and financial implications of their donation, with donors being concerned that they would be held legally responsible for a child they did not intend to be a father to and being chased for financial support from the Child Support Agency. Such concerns highlight that some donors are well aware of the legal risks they undertake when donating in the unregulated online market. Sperm bank donors most commonly expressed concerns about the identity-release nature of their donation and the impact of future contact with the child. Although sperm bank donors are clear about their role as a sperm donor until the child turns 18 years of age, they have concerns about the impact of their donation, both for themselves and any family they may have at that time, when their identity can be released to the donor-conceived child and they may try to make contact with them. Sperm bank donors would benefit from advice and support in navigating contact with donor-conceived offspring in the run up to 2023 when this becomes a possibility. In addition, some online donors may benefit from being incorporated into the regulated sector in order to avoid the legal and financial concerns they face in the online market. Donors who wish to donate anonymously are clearly not suitable as HFEA registered donors but is there scope to encourage known donation in the regulated sector? One way could be to encourage sperm donors and recipients to connect with each other online but then attend a clinic for treatment as a known donor. Freeman, (2016b) noted that a small minority (5.7%) of online donors who had conceived children had donated through a clinic but that a greater proportion showed a preference for clinic donation. Another possibility is to offer varying levels of information exchange between recipients and donors in the regulated sector so that donors and recipients have more control over levels of communication and the extent of contact they desire, a motivation currently driving both donors and recipients to use connection sites (Freeman, 2016b: Jadva, 2018; Bossema et al., 2014; Woestenburg et al., 2016). Indeed, Crawshaw et al. (2016) reported that some clinics, in and outside the USA, are already offering exchange of non-identifying or identifying information and ‘in person’ meetings, delivered either at the time of donation, whilst the child is growing up or once the child has reached 18 years. Moreover, in Victoria, Australia, parents of donor-conceived children can apply for access to their donor’s identity through the State’s central register and can do so upon their child’s birth. This information can be released to the parent if the donor consents (Kelly and Dempsey, 2017). Systems to allow both donor and recipients more control and choice over information exchange could be beneficial to the regulated sector in the UK. Incorporating online donors into the regulated system would not only increase the number of donors in the regulated system but also provide these men, who would have otherwise donated online, with the legal protection that regulation entails.

The present study had a number of limitations. Although data were collected from large groups of donors, donors were recruited from one connection site and one sperm bank only. Despite Pride Angel being one of the largest and most well-known connection sites in the UK, and The London Sperm Bank the largest sperm bank in the UK, these findings may not be representative of all sperm donors donating in the regulated sector, nor those donating in the unregulated sector online. Specifically, these are a particular type of online donor—men who are donating through a subscription based connection site where donors have to buy credits to message recipients, rather than through openly accessed Facebook groups etc. Freeman, (2016b) noted differences between donors on Pride Angel whose sperm had been used to conceive children and those whose sperm had not yet been used. They suggest that recipients may ‘filter out’ donors who are less well intentioned. The online donors in this study perhaps represent the ‘ideal’ online donor and may not be representative of the motivations and expectations of men donating in the unregulated sector more generally. Moreover, the online donors had all donated sperm prior to the time of the administration of the online donor questionnaire in 2014. The field of online donation has grown rapidly in recent years and therefore the online donors reported upon in this study may not be representative of men donating sperm online today.

The questionnaire nature of the data collection, with some ‘forced response’ questions, also has certain limitations for the conclusions that can be drawn. Respondents may have felt compelled to choose socially desirable answers, for example rating ‘I want to help others’ highly when asked about their motivations. Moreover, as Mohr (2014) argues, ‘Becoming a sperm donor involves men as whole persons, not only as actors who make decisions that they can also account for’. Further in-depth, qualitative studies that examine the sperm donor’s own narratives are needed in order to further explore the issues that this questionnaire based comparative study has raised.

Despite these limitations, this study provides valuable empirical insights that have relevance for policy and practice. It has highlighted the similarities and differences between online and sperm bank donors and the plurality of meanings which being a sperm donor can entail. It has shown that the boundary between regulated and unregulated donation may not be distinct, emphasising the importance of considering ways to incorporate some online donors into the regulated system—both to increase the number of sperm donors available at clinics and to provide legal protection and support for donors. Ultimately, this study stresses the need for further, in-depth, longitudinal studies of both HFEA registered and online donors, exploring how informal donor arrangements work out over time, and how both groups think about, and manage, information exchange and contact with the offspring conceived through their donation.

## References

[ref1] Ahuja K (2011) ‘If it ain’t broke, don’t fix it: why the HFEA should leave the gamete donation policy alone.’ Available at http://www.bionews.org.uk/page_107736.asp

[ref2] Blyth E, Frith L The Uk’s gamete donor ‘crisis’ – a critical analysis. Crit Soc Policy 2008;28:74–95.

[ref3] Bossema ER, Janssens PMW, Treucker GL, Landwehr F, Duinen K van, Nap AW, Geenen R An inventory of reasons for sperm donation in formal versus informal settings. Hum Fertil 2014;17:21–27. 10.3109/14647273.2014.881561 24575758

[ref4] Crawshaw M, Daniels K, Adams D, Bourne K, Hoof JAP van, Kramer W, Pasch L, Thorn P Emerging models for facilitating contact between people genetically related through donor conception: a preliminary analysis and discussion. Reproductive Biomedicine and Society Online 2016;1:71–80. 10.1016/j.rbms.2015.10.001 PMC6001351 29911188

[ref5] Crocker (2016).’Super dad sperm donor has 800 children and becomes a father once a week’. The Mirror, 13th January 2016 Available at https://www.mirror.co.uk/news/uk-news/super-dad-sperm-donor-800-7170289

[ref6] Day M Number of sperm donors rises in UK despite removal of anonymity. BMJ 2007;334:971. 17493999 10.1136/bmj.39206.514132.DB PMC1867873

[ref7] Daniels K, Blyth E, Crawshaw M, Curson R Short communication: previous semen donors and their views regarding the sharing of information with offspring. Hum Reprod 2005;20:1670–1675. 15760955 10.1093/humrep/deh839

[ref8] Dempsey D Conceiving and negotiating reproductive relationships: lesbians and gay men forming families with children. Sociology 2010;44:1145–1162.

[ref10] Freeman T, Jadva V, Slutsky J Sperm donors limited: psychosocial aspects of genetic connections and the regulation of offspring numbers In: Golombok S, Scott R, Appleby JB, Richards M, Wilkinson S (eds). Regulating Reproductive Donation. Cambridge University Press, 2016a.

[ref9] Freeman T, Jadva V, Tranfield E, Golombok S Online sperm donation: a survey of the dempgraphic characteristics, motivations, preferences and experiences of sperm donors on a connection website. Hum Reprod2016b;31:2082–2089. 27412344 10.1093/humrep/dew166 PMC4991659

[ref11] Frith L, Blyth E, Farrand A UK gamete donor’s reflections on the removal of anonymity: implications for recruitment. Hum Reprod 2007;22:1675–1680. 17449513 10.1093/humrep/dem061

[ref12] Ghuman N, Mair E, Pearce K, Choudhary M Does age of the sperm donor influence live birth outcome in assisted reproduction? Hum Reprod 2016;31:582–590. 26762315 10.1093/humrep/dev331

[ref13] Goldberg AE, Allen K Donor, dad, or … ? Young adults with lesbian parents' experiences with known donors. Fam Process 2013;52:338–350. 23763691 10.1111/famp.12029

[ref14] Graham S, Mohr S, Bourne K Regulating the ‘good’ donor: the expectations and experiences of sperm donors in Denmark and Victoria, Australia In: Golombok S, Scott R, Appleby JB, Richards M, Wilkinson S (eds). Regulating Reproductive Donation. Cambridge University Press, 2016.

[ref15] Harper J, Jackson E, Spoelstra-Witjens L, Reisel D Using an introduction website to start a family: implications for users and health practitioners. Reproductive Biomedicine and Society Online 2017;4:13–17. 29796427 10.1016/j.rbms.2017.02.001 PMC5957093

[ref16] HFEA. Disclosure of Donor Information. Regulations. London: HFEA, 2004.

[ref17] HFEA (2013). Egg and sperm donation in the UK: 2012-2013. Available at https://www.hfea.gov.uk/about-us/publications/research-and-data/

[ref18] HFEA. (2014). Using donated sperm, eggs or embryos in you treatment. Available at https://www.hfea.gov.uk/treatments/explore-all-treatments/using-donated-eggs-sperm-or-embryos-in-treatment/.

[ref19] HFEA. (2018). ‘Donating your sperm’. Available at https://www.hfea.gov.uk/donation/donors/donating-your-sperm/

[ref20] HFEA. (2019). ‘Sperm donation and the law – for donors’. Available at https://www.hfea.gov.uk/donation/donors/donating-your-sperm/sperm-donation-and-the-law-for-donors/

[ref21] Jadva V, Freeman T, Tranfield E, Golombok S Why search for a sperm donor online? The experiences of searching for and contacting sperm donors on the internet. Hum Fertil 2018;21:112–119. 10.1080/14647273.2017.1315460 PMC5951149 28449623

[ref22] Kelly F, Dempsey D Experiences and motives of Australian single mothers by choice who make early contact with their donor’s relatives. Med Law Rev 2017;24:571–590. 10.1093/medlaw/fww038 PMC5388365 28137771

[ref23] Kirkman M, Bourne K, Fisher J, Johnson L, Hammarberg K Gamete donors’ expectations and experiences of contact with their donor offspring. Hum Reprod 2014;29:731–738. 24549216 10.1093/humrep/deu027 PMC3949499

[ref24] Lea L. (2016). ‘UK’s national sperm bank stops recruiting donors’, *BBC News,* 27 October 2016. Last accessed 24 09 2018.

[ref25] Mohr S Beyond motivation: on what it means to be a sperm donor in Denmark. Anthropol Med 2014;21:162–173. 25175292 10.1080/13648470.2014.914806 PMC4200559

[ref26] Pennings G The internal coherence of donor insemination practice: attracting the right type of donor without paying. Hum Reprod 1997;12:1842–1844. 9363692 10.1093/oxfordjournals.humrep.a019604

[ref27] Ravelingien A, Proovost V, Pennings G Creating a family through connection websites and events: ethical and social issues. Reprod Biomed Online 2016;33:522–528. 27502067 10.1016/j.rbmo.2016.07.004

[ref27a] Riggs D Lesbian mothers, gay sperm donors, and community: Ensuring the wellbeing of children and families. Health Sociology Review 2008;17:226–234.

[ref27b] Riggs D, Russel L Characteristics of men willing to act as sperm donors in the context of identity-release legislation. Human Reproduction 2011;26:266–272. 21088014 10.1093/humrep/deq314

[ref28] Van den Broeck U, Vandermeeren M, Vanderschueren D, Enzlin P, Demyttenaere K, D’Hooghe T A systematic review of sperm donors: demographic characteristics, attitudes, motives and experiences of the process of sperm donation. Hum Reprod Update 2013;19:37–51. 23146866 10.1093/humupd/dms039

[ref29] Wilkinson S Gamete donor motives, payment and child welfare In: Golombok S, Scott R, Appleby JB, Richards M, Wilkinson S (eds). Regulating Reproductive Donation. Cambridge University Press, 2016.

[ref30] Whyte S, Savage DA, Torgler B Online sperm donors: the impact of family, friends, personality and risk perception on behaviour. Reprod Biomed Online 2017;35:723–732. 28951001 10.1016/j.rbmo.2017.08.023

[ref31] Whyte S Clinical vs exclusively online sperm donors: what’s the difference? J Reprod Infant Psychol 2019;37:3–12. 30375886 10.1080/02646838.2018.1540864

[ref32] Woestenburg NOM, Winter HB, Janssens PMB What motivates men to offer sperm donation via the internet? Psychol Health Med 2016;21:424–430. 26339943 10.1080/13548506.2015.1081702

